# Antipsychotic prescription amongst the elderly: Descriptive and analytical study

**DOI:** 10.1192/j.eurpsy.2023.1998

**Published:** 2023-07-19

**Authors:** W. Bouali, R. Omezzine Gniwa, S. Younes, M. Kacem, L. Zarrouk

**Affiliations:** 1 Psychiatrie; 2 Family medicine, Faculty of Medicine of Monastir, Mahdia, Tunisia

## Abstract

**Introduction:**

Antipsychotics are frequently used to treat certain delusional, psychotic and behavioral symptoms in the elderly. However, the data in the literature show a great variability in the practices of different teams as well as numerous misuses in the use of this therapeutic class.

**Objectives:**

The aims of this work were to evaluate the prescription of antipsychotics in the elderly admitted in a psychiatric department and to compare them with the Information in the literature.

**Methods:**

This was a retrospective and descriptive study carried out in the Psychiatry Department at Mahdia Hospital. We included all patients aged 65 years and older, admitted between January 2016 and December 2021 and having received antipsychotic treatment.

**Results:**

Our sample consisted of 53 patients with a mean age of 69.8 years with a standard deviation of 4.2. The sex ratio (M/W) was 2.7. The most common diagnoses in our sample were schizophrenia and dementia with rates of 31.8% and 27.3% of cases respectively. Regarding antipsychotic treatment, 34.1% received first generation oral antipsychotic treatment (AP1G), 31.8% received second-generation oral antipsychotic treatment (AP2G), 27.3% received a combination of AP1G and AP2G and 6.8% received a long-acting injectable antipsychotic. More than a quarter of our patients (34.1%) reported adverse events due to antipsychotic treatment.

**Conclusions:**

The results of our study highlighted various indications for which an antipsychotic treatment was prescribed in an elderly person despite an often poor and multi-medicated health status, to which side effects were added.

**Disclosure of Interest:**

None Declared

